# Thanks to all those who reviewed for *Biological Procedures Online* in 2015

**DOI:** 10.1186/s12575-016-0035-0

**Published:** 2016-03-03

**Authors:** Shulin Li

**Affiliations:** Department of Pediatrics–Research, Unit 0853, The University of Texas MD Anderson Cancer Center, Austin, USA

## Abstract

A peer-reviewed journal would not survive without the generous time and insightful comments of the reviewers, whose efforts often go unrecognized. Although final decisions are always editorial, they are greatly facilitated by the deeper technical knowledge, scientific insights, understanding of social consequences, and passion that reviewers bring to our deliberations. For these reasons, the Editor-in-Chief and staff of *Biological Procedures Online* warmly thank the 30 reviewers whose comments helped to shape the journal, for their invaluable assistance with review of manuscripts in Volume 17 (2015).

**Ergul Belge Kurutas**

Turkey

**Shantanu Bhatt**

USA

**Xiaolei Chen**

USA

**Wim D'Haeze**

USA

**Sofia Duarte**

Portugal

**Christine Ebel**

France

**David Gewirtz**

USA

**Eduardo Gilglioni**

Brazil

**Brenda Gonzalez-Hernandez**

Mexico

**S Kavimani Kavimani**

India

**Sandro Keller**

Germany

**Yan Luo**

USA

**George McNamara**

USA

**Sang Meixiang**

USA

**Meral Miraloglu**

Turkey

**Tapan Mohanta**

Korea, Republic of

**Craig Nunemaker**

USA

**Toshifumi Ozaki**

Japan

**Satyabrata Pany**

USA

**M D Shahjahan**

Bangladesh

**Robert Silverman**

USA

**James Sturgis**

France

**Masatoshi Tagawa**

Japan

**Roger Y Tsien**

USA

**Liyang Wang**

China

**Wei Xiawei**

China

**Xuehong Xu**

China

**Shaomian Yao**

USA

**Swee Keong Yeap**

Malaysia

**Ying Yu**

China

